# Aspirin use and bleeding events during thrombocytopenia after autologous stem-cell transplantation for multiple myeloma

**DOI:** 10.3389/fonc.2023.1168120

**Published:** 2023-04-27

**Authors:** Nina Rosa Neuendorff, Boryana Boshikova, Lutz Frankenstein, Marietta Kirchner, Christian Rohde, Hartmut Goldschmidt, Norbert Frey, Carsten Müller-Tidow, Karin Jordan, Sandra Sauer, Maike Janssen

**Affiliations:** ^1^ Department of Medicine V–Hematology, Oncology and Rheumatology, University Hospital Heidelberg, Heidelberg, Germany; ^2^ Department of Hematology and Stem-Cell Transplantation, University Hospital Essen, Essen, Germany; ^3^ Clinic for Cardiology and General Internal Medicine, Städtisches Klinikum Solingen gemeinnützige GmbH, Solingen, Germany; ^4^ Department of Medicine III–Cardiology, Angiology and Intensive Care, University Hospital Heidelberg, Heidelberg, Germany; ^5^ Institute of Medical Biometry (IMBI), University Hospital Heidelberg, Heidelberg, Germany; ^6^ Klinikum Ernst von Bergmann–Department for Hematology, Oncology and Palliative Care, Potsdam, Germany

**Keywords:** multiple myeloma, autologous stem-cell transplantation, antiplatelet therapy, aspirin, bleeding, cardio-vascular events

## Abstract

**Background:**

In patients with cardiovascular (CV) comorbidities that necessitate antiplatelet therapy (APT), its optimal management during chemotherapy-induced thrombocytopenia remains elusive, as the risk of bleeding has to be balanced against the risk of CV events. The purpose of this study was to assess the risk for bleeding with APT during thrombocytopenia in patients with multiple myeloma undergoing high-dose chemotherapy and subsequent autologous stem-cell transplantation (ASCT) with and without acetylsalicylic acid (ASA) as comedication.

**Methods:**

We assessed patients who underwent ASCT at the Heidelberg University Hospital between 2011 and 2020 for bleeding events, management strategies for ASA intake during thrombocytopenia, transfusion requirements, and the occurrence of CV events.

**Results:**

There were 57/1,113 patients who continued ASA until at least 1 day after ASCT; thus, a continuous platelet inhibition during thrombocytopenia was assumed. Most of the patients (41/57) continued ASA until they had a platelet count of 20–50/nl. This range reflects the kinetics of thrombocytopenia and nondaily measurements of platelets during ASCT. A tendency toward a higher risk for bleeding events in the ASA group was demonstrated (1.9% (control group) *vs*. 5.3% (ASA), p = 0.082). The risk factors for bleeding in multivariate analysis were the duration of thrombocytopenia < 50/nl, a history of gastrointestinal bleeding, and diarrhea. The factors predicting the duration of thrombocytopenia were age >60 years, a hematopoietic stem-cell transplantation comorbidity index ≥3, and an impaired bone marrow reserve at admission. CV events occurred in three patients; none of them took ASA or had an indication for APT.

**Conclusions:**

The intake of ASA until thrombocytopenia with a platelet count of 20–50/nl appears safe, although an elevated risk cannot be excluded. If ASA is indicated for the secondary prevention of CV events, the evaluation of risk factors for bleeding and a prolonged time of thrombocytopenia before conditioning is crucial to adapt the strategy for ASA intake during thrombocytopenia.

## Introduction

Cardiovascular (CV) disease is common in patients with cancer (1, 2). Due to impaired bone marrow function and more often therapy related, cancer patients frequently present thrombocytopenia with a platelet count (PLC) ≤50 × 10^9^/L which does not preclude from CV events (3, 4). Acetylsalicylic acid (ASA) has an outstanding role for the prevention of major CV events (MACE) (5), including nonfatal stroke, myocardial infarction, and CV death. Many strategies to handle ASA during thrombocytopenia exist without appropriate evidence as data are lacking to what extent ASA increases the bleeding risk in these scenarios.

In daily practice, ASA is frequently withheld during thrombocytopenia National Cancer Institute Common Terminology Criteria for Adverse Events (CTCAE) III–IV° (PLC < 25–50 × 10^9^/L). However, the discontinuation of ASA leads to the rebound reactivity of platelets caused by increasing thromboxane A2 (TXA2) levels (6). MACE after the discontinuation of ASA are as frequent as one additional event in every 36 patients (7) in studies outside an oncological setting (7–13).

Bleeding events (BLEDs) during thrombocytopenia (14) or antiplatelet therapy (APT) (15) can be life-threatening. The risk for serious BLEDs is not necessarily related to the severity of thrombocytopenia (16, 17), although a critical PLC of 5–7 × 10^9^/L was suggested as required to maintain endothelial integrity to prevent bleeding (18, 19). Although life-threatening bleeding during autologous hematopoietic stem-cell transplantation (ASCT) is rare (14, 20), less severe BLEDs can potentially lead to patient discomfort, prolonged in-patient stay, increased healthcare costs, or further complications. In a recent trial on different platelet transfusion (PLTX) strategies, 19% of ASCT patients developed mild to moderate BLEDs (20). A recent large prospective randomized trial assessed a prophylactic PLTX approach with a morning PLC of < 10 × 10^9^/L in comparison to a therapeutic approach in case of apparent bleeding signs in patients undergoing ASCT or intensive chemotherapy for acute leukemia. The therapeutic approach led to a significant reduction of provided PLTX without increasing the risk for major BLEDs in patients undergoing ASCT (21). Thus, a therapeutic approach appears to be safe. Moreover, trials and retrospective data analysis that assessed the effect of PLTXs in patients with intracranial or gastrointestinal (GI) bleeding upon APT and a mostly normal PLC found even a negative impact on death and/or disability (22–24). Thus, there is no evidence that fosters a liberal transfusion approach so far.

To evaluate the risk of bleeding during thrombocytopenia and concomitant APT, we reviewed BLEDs in a large cohort of multiple myeloma patients with and without APT undergoing ASCT.

## Materials and methods

### Ethical considerations

The study design and data acquisition were approved by the institutional review board of the University Heidelberg, Germany, No S-721/2018, and the study was performed in accordance with the Declaration of Helsinki.

### Study population

Patients undergoing ASCT for multiple myeloma at the in-patient service of the University Hospital Heidelberg between 2011 and February 2020 were included. Patients undergoing their ASCT as outpatients, being on dual APT or therapeutic anticoagulation, were excluded. Patient characteristics and the course of ASCT were captured by the review of electronical medical records (EMRs).

### Study definitions and outcomes

The primary outcome was the occurrence of BLEDs during hospital stay. These were staged according to CTCAE, version 5.0 (National Cancer Institute, Bethesda, MD), the Bleeding Severity Measurement Scale (BSMS) (25), and the World Health Organization (WHO) bleeding scale. In-depth discussion and rationale for the use of these bleeding scales are summarized and discussed in the **Supplementary Material** (1.1 **Supplementary Methods**, 1.1.1 Grading of bleeding events, their definitions, and rationale).

Secondary outcomes included the number of PLTXs, the time to the engraftment of platelets, and MACE. For the purpose of this study, MACE was defined as nonfatal stroke, nonfatal myocardial infarction, and CV death.

### Management of antiplatelet therapy and patient allocation

ASA therapy at a dose of 75–100 mg during ASCT was managed at individual physicians’ discretion. ASA was regarded as having an efficient antiplatelet function during thrombocytopenia if at least one dose of ASA after the reinfusion of stem cells (day 0) was provided as the autologous stem-cell preparation contained platelets and plasma potentially reverting the ASA effect. Furthermore, it was assumed that all platelets at this point were sufficiently inhibited and no turnover took place until hematopoietic recovery. ASA was usually recommenced with the recovery of PLC >20–50 × 10^9^/L. Patients who stopped ASA intake between hospital admission and earlier than 1 day after stem-cell reinfusion (day +1) were excluded from main analyses because neither ASA efficacy could not be assumed nor excluded. BLEDs and the patient characteristics of this group were separately assessed and analyzed to exclude a selection bias.

### Center-specific transfusion management during autologous stem-cell transplantation

During the study period, two major transfusion strategies existed at our center (1): prophylactic PLTX if morning PLC < 10 × 10^9^/L and (2) PLTX restricted to signs of bleeding as recently described (21), based on the treating physician’s discretion. Because minor bleeding signs were not always documented in the EMR, a prophylactic versus therapeutic approach could not assigned to every provided PLTX and was therefore not further integrated into analyses.

There were 1 to 2 units of packed red blood cell (PRBC) transfusions that were usually provided in case of hemoglobin ≤ 70 or 80 g/L with symptomatic anemia.

### Statistical analysis

Categorical variables were summarized by number and percentage, and continuous variables were summarized by mean, standard deviation, and range (minimum/maximum). Continuous variables were compared between ASA and the control group (or as stated otherwise) using Welch’s t-test for unequal variances and categorial variables by the chi-square test. Univariate logistic regression models were built to test the association between BLEDs or PLTX requirements, respectively, and all relevant patient characteristics, clinical, and laboratory variables to determine the potential predictors of BLEDs or PLTX requirements. For PLTX requirements, patients with BLEDs were excluded from the analysis to avoid bias due to increased PLTX requirements during bleeding. Multivariable logistic regression models using stepwise estimation by backward and forward selection were then generated from variables with *p* ≤ 0.05 from the univariate models and known/expected factors to be associated with BLEDs or PLTX requirements. Firth’s logistic regression was used to confirm results for BLEDs as logistic regression could be biased by unbalanced predictors.

A linear regression model to assess risk factors for a prolonged duration of thrombocytopenia in patients without BLEDs to avoid a bias for increased transfusions during bleeding was built. Additionally, two outlier cases with an unusual requirement were excluded to avoid a bias for this analysis.

The rationale and sample size calculation for a randomized controlled trial to evaluate different ASA discontinuation strategies are described in the **Supplement Material** (1.1 **Supplementary Methods**, 1.1.2 Design of a randomized controlled trial to assess different APT strategies, and **Supplementary Figure S1**).

All statistical analyses were performed in SPSS version 28.0.0.0 and SAS version 9.4. Figures were created with R-studio version 4.1.2, Excel, and Biorender.

## Results

### Study population and patient characteristics

There were 13 patients who were excluded as they paused ASA intake earlier than 1 day after stem-cell reinfusion (day +1); therefore, neither ASA efficacy could be expected nor excluded (the patient characteristics of this group are depicted in **Supplementary Table S1**). Thus, 1,113 patients were included into analysis. Baseline patient characteristics are summarized in **Table 1**. The patient characteristics of those who took ASA in comparison to those without (control group) revealed significant differences in age, gender, and the hematopoietic stem-cell transplantation comorbidity index (HCT-CI). Furthermore, patients in the ASA group had more CV comorbidities as expected. All other characteristics were similar between groups.

**Table 1:** Patient characteristics.

Characteristics	All (n = 1,113)	ASA cohort (n = 57)	Control cohort (n = 1,056)	p-value
**Age, mean ± SD (range)** **≥65 years (%)**	58.9 ± 8.36 (28–76) 332 (29.8%)	61.12 ± 8.07 (36–75) 26 (45.6%)	58.79 ± 8.36 (28–76) 306 (29%)	0.04 0.017
**Female, N (%)**	430 (38.6%)	11 (19.3%)	419 (39.7%)	< 0.001
**HCT-CI, mean ± SD (range)** **≥3, N (%)**	1.6 ± 1.99 (0–11) 284 (25.5%)	2.95 ± 1.94 (0–7) 31 (54.4%)	1.53 ± 1.96 (0–11) 253 (24%)	< 0.001 < 0.001
**First ASCT, N (%)**	733 (65.9%)	41 (71.9%)	692 (65.5%)	0.304
**Second ASCT, N (%)**	376 (33.8%)	16 (28.1%)	360 (34.1%)	
**Third ASCT, N (%)**	4 (0.4%)	0 (0%)	4 (0.4%)	
**Transfused CD34+ cells × 10^6^/kg mean ± SD (range)**	370.48 ± 163.38 (74–1,404)	365.93 ± 130.73 (202–836)	370.73 ± 165.01 (74–1,404)	0.82
Concomitant medications during ASCT				
**SSRI, N (%)**	59 (5.3%)	4 (7%)	55 (5.2%)	0.55
**PPI, N (%)**	1,098 (98.7%)	57 (100%)	1,041 (98.6%)	0.365
**GCS, N (%)**	6 (0.5%)	1 (1.8%)	5 (0.5%)	0.471
Laboratory measurements at admission				
**Hemoglobin [g/dl], mean ± SD (range)** **< 10 g/dl, N (%)** **< 8 g/dl, N (%)**	11.77 ± 1.43 (7.8–18) 84 (7.5%) 2 (0.2%)	11.86 ± 1.36 (9–15.7) 4 (7%) 0 (0%)	11.77 ± 1.44 (7.8–18) 80 (7.6%) 2 (0.2%)	0.63 0.91 0.74
**Platelets/nl, mean ± SD (range)** **<100 × 10^9^/L, N (%)** **<50 × 10^9^/L, N (%)**	234.4 ± 72.9 (22–617) 22 (2%) 3 (0.3%)	234.39 ± 74.60 (135–607) 0 (0%) 0 (0%)	234.39 ± 72.84 (22–617) 22 (2.1%) 3 (0.3%)	0.99 < 0.001 0.69
**PTT [%], mean ± SD (range)** **<70%, N (%)**	102.93 ± 12.19 (55–125) 11 (1%)	103.61 ± 11.53 (71–125) 0 (0%)	102.84 ± 12.23 (55–125) 11 (1%)	0.66 0.62
**aPTT, mean ± SD (range)** **>40, N (%)**	24.59 ± 5.47 (12–120) 12 (1.1%)	26.05 ± 13.00 (20–120) 1 (1.8%)	24.51 ± 4.73 (12–120) 11 (1%)	0.61 0.61
**History of GI bleeding, N (%)**	12 (1.1%)	3 (5.3%)	9 (0.9%)	0.15
**History of CNS bleeding, N (%)**	6 (0.5%)	0 (0%)	6 (0.6%)	0.56
**History of retina bleeding, N (%)**	0 (0%)			
Adverse events during ASCT				
**Neutropenic fever, N (%)**	1,001 (89.9%)	55 (96.5%)	946 (89.6%)	0.023
**Pulmonary infection, N (%)**	127 (11.4%)	11 (19.3%)	116 (11.0%)	0.126
**Sepsis, N (%)**	113 (10.2%)	7 (12.3%)	106 (10.0%)	0.585
**Diarrhea, N (%)**	264 (23.7%)	15 (26.3%)	249 (23.6%)	0.637
Cardiovascular comorbidities				
**CAD**	70 (6.3%)	36 (63.2%)	34 (3.2%)	< 0.001
**AF**	31 (2.8%)	2 (3.5%)	29 (2.7%)	0.73
**PAD**	12 (1.1%)	11 (19.3%)	1 (0.1%)	< 0.001
**TIA**	5 (0.4%)	1 (1.8%)	4 (0.4%)	0.439
**Stroke**	10 (0.9%)	8 (14%)	2 (0.2%)	0.004
**PFO**	4 (0.45)	0 (0%)	4 (0.4%)	0.624
**CRAO**	5 (0.4%)	2 (3.5%)	3 (0.3%)	0.196
**PAH**	15 (1.3%)	1 (1.8%)	14 (1.3%)	0.78
**HF**	34 (3.1%)	8 (14%)	26 (2.5%)	0.016
Prior TE event				
**DVT** **PE**	75 (6.7%) 33 (3%)	1 (1.8%) 1 (1.8%)	74 (7%) 32 (3%)	0.008 0.58
Cardiovascular risk factors				
**Hyperlipidemia**	63 (5.7%)	25 (43.9%)	38 (3.6%)	< 0.001
**Diabetes mellitus**	96 (8.6%)	12 (21.1%)	84 (8%)	0.021
**History of smoking** **Active smoking**	148 (13.3%) 45 (4%)	16 (28.6%) 5 (8.8%)	132 (12.5%) 40 (3.8%)	0.013 0.198
**Hypertension**	335 (30.1%)	35 (61.4%)	300 (28.4%)	< 0.001
**OSA**	23 (2.1%)	1 (1.8%)	22 (2.1%)	0.865
Indications for ASA intake				
**Primary prevention** **Secondary prevention**	8 (0.7%) 82 (7.4%)	5 (8.8%) 52 (91.2%)	3 (0.3%) 30 (2.8%)	< 0.001 < 0.001

AF, atrial fibrillation; ASA, acetylsalicylic acid; ASCT, autologous stem-cell transplantation; CAD, coronary artery disease; CNS, central nervous system; CRAO, central retinal artery occlusion; DVT, deep vein thrombosis; GCS, glucocorticosteroids; GI, gastrointestinal; HF, heart failure; OSA, obstructive sleep apnea; PAD, peripheral artery disease; PAH, pulmonary arterial hypertension; PE, pulmonary embolism; PFO, patent foramen ovale; PTT, prothrombin time; SD, standard deviation; TE, thromboembolic event; TIA, transient ischemic attack.

### Management of acetylsalicylic acid intake during thrombocytopenia

For 57 patients, continuous ASA efficacy during thrombocytopenia was assumed as ASA was continued until at least 1 day after ASCT. Of those, the majority (41/57) continued ASA until PLC 20–50 × 10^9^/L (11/57 patients stopped with PLC < 70–80 × 10^9^/L, 28/57 with PLC < 50 × 10^9^/L, 13/57 with PLC < 20–30 × 10^9^/L, and 2 with PLC < 10 × 10^9^/L. Only one patient continued ASA throughout thrombocytopenia, and for 2/57 patients, no clear strategy could be defined). The mean day of last ASA intake after ASCT was day +4.89 ± 1.37 (range: day 2–7), the mean days of ASA discontinuation were 9.39 ± 6.02 (range: 0–42 days; 95% confidence interval (CI) [8, 11.19]).

### Bleeding events

**Figure 1:**
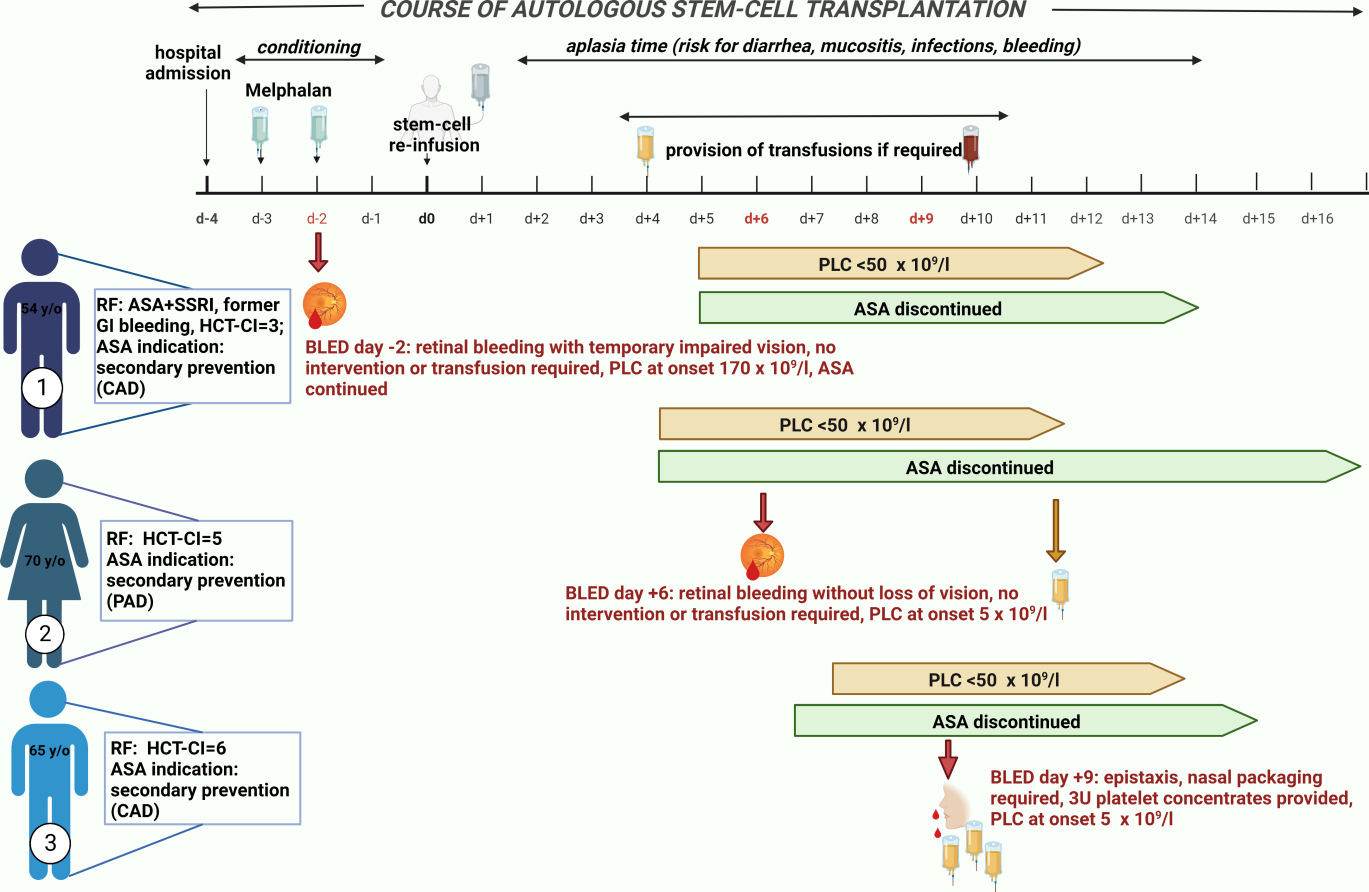
Bleeding events in patients taking acetylsalicylic acid (ASA). Three patients who took ASA developed a bleeding event. Patient 1 suffered from a retinal hemorrhage on day −2. He took ASA on the day of bleeding onset but still had a normal platelet count of 170 × 10^9^/L. As risk factors for bleeding, he took a selective serotonin reuptake inhibitor (SSRI) in addition to ASA and had a former gastrointestinal (GI) bleeding and a hematopoietic stem-cell transplantation comorbidity index (HCT-CI) of 6. The indication for ASA was secondary preventive due to coronary heart disease. During bleeding, he had a temporarily impaired vision but without any need of intervention or transfusion. He reported no sequelae. Patient 2 suffered also from a retinal hemorrhage on day +6 after autologous stem-cell reinfusion during a thrombocytopenia of 5 × 10^9^/L. ASA was discontinued 2 days before due to onset of thrombocytopenia < 50 × 10^9^/L. She did not receive any platelet transfusion (PLTX) prior to bleeding onset; thus, continuous full platelet inhibition by ASA could be expected. She developed only very mild symptoms; thus, no intervention or transfusion was required and no sequelae were reported. Her indication for ASA intake was peripheral artery disease and, as risk factors for bleeding, only an HCT-CI of 5 can be described. Patient 3 developed epistaxis on day +9 after stem-cell reinfusion during a thrombocytopenia of 5 × 10^9^/L. Nasal packaging by an ear–nose–throat specialist was performed, and he received 3 units of PLTXs. ASA was discontinued 3 days prior to bleeding onset without any PLTX in the meantime; thus, continued platelet inhibition could be assumed. As risk factors for bleeding, an HCT-CI of 6 existed and the indication for ASA intake was secondary preventive due to coronary heart disease. All three bleeding events were single events without ongoing bleeding after first onset. *ASA, acetylsalicylic acid; BLED, bleeding events; CAD, coronary artery disease; GI, gastrointestinal; HCT-CI, hematopoietic stem-cell transplantation comorbidity index; PAD, peripheral artery disease; PLC, platelet count; RF, risk factor; SSRI, selective serotonin reuptake inhibitor*.

A clinically meaningful, documented BLED occurred in 23 patients (2.1%). Of these 23 patients, three patients were in the ASA group. Two of them discontinued ASA before the onset of a BLED, although a continued efficacy of APT was suspected. The third patient took ASA on the day of bleeding, but PLC was still normal. The clinical course of these three patients is depicted in **Figure 1**. No significant difference in bleeding incidence was demonstrated for patients receiving ASA, although a higher tendency was observed [1.9% (control group) *vs*. 5.3% (ASA), p = 0.082].

**Figure 2:**
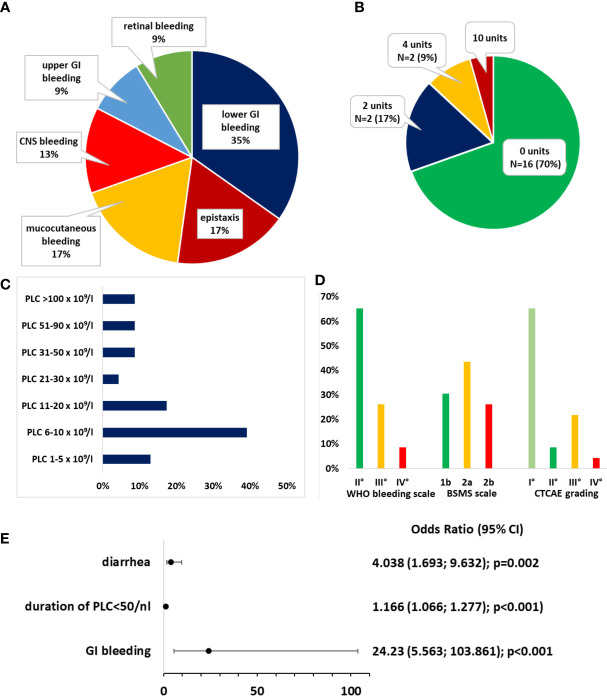
Bleeding events. **(A)** Distribution of bleeding sites. **(B)** Distribution of transfusion requirements for packed red blood cells. **(C)** Distribution of platelet count (PLC) at bleeding onset. **(D)** Distribution of bleeding severity according to the World Health Organization (WHO) bleeding scale, Bleeding Severity Measurement Scale (BSMS), and NCI Common Terminology Criteria for Adverse Events (CTCAE). **(E)** Odds ratios (ORs) for the risk of bleeding. A binomial logistic regression model was generated after forward selection.

The mean PLC at bleeding onset was 30.87 × 10^9^/L ± 46.48 (range: 4–179 × 10^9^/L). There were 52.2% (N = 12) of BLEDs that occurred during PLC ≤ 10 × 10^9^/L. In total, 82.6% of BLEDs were documented during PLC ≤ 50 × 10^9^/L (**Figure 2C**). The median day of bleeding onset was day +6 after ASCT (range: day −2 to +40). There were 16/23 BLEDs (69.6%) that occurred during a documented infection. The majority constituted lower GI bleedings (34.8%) (**Figure 2A**).

A specialist consultation (e.g., neurology and urology) was initiated in 8/23 patients, an endoscopy without further intervention was performed in two, and three patients underwent nasal packing to control epistaxis, which was the only intervention that was performed. The transfusion requirements and gradings of bleedings according to the WHO bleeding scale, CTCAE grading, and the BSMS are depicted in **Figures 2B, D**. In 16 patients, the BLED was deemed clinically significant according to the BSMS (comprising grade 2a/b). No relevant sequelae after hospital discharge were documented.

Of note, among patients who were excluded from analysis as they paused ASA intake earlier than 1 day after stem-cell reinfusion (day +1), therefore, no ASA efficacy was expected, one more patient developed a retinal hemorrhage. Thus, all three documented retinal hemorrhages occurred in patients with ASA. Further details on BLEDs are summarized in **Supplementary Table S2**.

### Risk factors for bleeding

We further assessed the risk factors for BLEDs. Hypertension, the duration of PLC ≤ 50 × 10^9^/L, diarrhea, a history of GI bleeding, and the HCT-CI score were significantly (but weakly) associated with BLEDs (data not shown). The odds ratio (OR) for ASA intake was 2.78 (95% CI, [0.83, 9.98], p = 0.096). In multivariate binomial logistic regression, ASA intake as a risk factor for bleeding was also not significant (**Supplementary Figure S2A**). After multivariate binomial logistic regression by backward selection, only the duration of PLC ≤ 50 × 10^9^/L (OR = 1.166, 95% CI, [1.066, 1.277]; p < 0.001), diarrhea (OR = 4.038, 95% CI, [1.693, 9.632], p = 0.002), and a history of GI bleeding (OR = 24.230, 95% CI, [5.653, 103.861], p < 0.001) remained significantly associated with bleeding risk (**Figure 1E**). This binomial logistic regression model was statistically significant, χ² (3) = 31.500, p < 0.001. Goodness-of-fit as assessed using Hosmer–Lemeshow test indicated a good model fit, χ²(3) = 8.690, p > 0.050. The correlations between predictor variables were low (r < 0.70), indicating that multicollinearity was not a confounding factor in the analysis. As only few BLEDs occurred, results were confirmed with Firth regression to avoid biased results by disproportions among the groups. The complete model and Firth regression are depicted in **Supplementary Figure S2A/B**. The duration of PLC ≤ 10 × 10^9^/L was not associated with an increased bleeding risk (**Supplementary Figure S2C**).

### Platelet count and transfusion strategies

**Figure 3:**
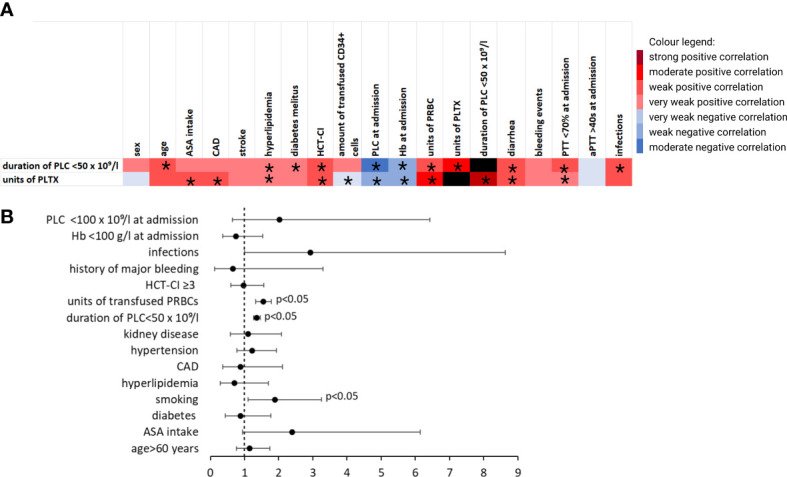
Transfusion requirements and duration of thrombocytopenia. **(A)** Heatmap showing Pearson’s correlation coefficient (phi coefficient for binary variables, respectively) visualizing factors that correlate with requirement of PLTX and duration of PLC < 50 × 10^9^/L. *indicates p < 0.05. **(B)** ORs for the requirement of >1 unit of platelets in patients without a bleeding event from multivariate logistic regression analysis after using stepwise estimation by backward and forward selection. *aPTT, activated partial thromboplastin time; ASA, acetylsalicylic acid; CAD, coronary artery disease; Hb, hemoglobin; HCT-CI, hematopoietic stem-cell transplantation comorbidity index; PLC, platelet count; PLTX, platelet transfusion; PRBC, packed red blood cells (in units); PTT, prothrombin time.*.

Basic transfusion requirements and PLCs are summarized in **Table 2**. A total of 477 (42.9%) patients received no PLTX at all; 481 (43.2%) received only one unit. Patients with ASA intake received significantly more units of PLTX (1.32 ± 1.42 *vs*. 0.78 ± 0.96, p < 0.001). No significant differences regarding the units of PRBCs or the duration of thrombocytopenia was demonstrated. The correlation between PLTX requirements and clinical factors is shown in **Figure 3A**. In binomial logistic regression analysis for PLTX requirements above the average of 0–1 unit of platelets, smoking, PRBCs, and the duration of PLC < 50 × 10^9^/L remained significant. ORs are depicted in **Figure 3B**. The binomial logistic regression model was statistically significant, χ²(15) = 198.747, p < 0.001. Goodness-of-fit as assessed by the Hosmer–Lemeshow test indicated a good model fit, χ²(15) = 7.326, p > 0.050. The correlations between predictor variables were low (r < 0.70). Since the duration of PLC < 50 × 10^9^/L showed a strong and clinical meaningful impact on PLTX requirements and BLEDs, risk factors for prolonged duration were further explored. The duration of PLC < 50 × 10^9^/L correlated significantly with age, PLC, hemoglobin, and prothrombin time (PTT) at admission, HCT-CI, hypertension, diarrhea, smoking, diabetes, hyperlipidemia, and infections (**Figure 3A**). The same factors were significant in univariant linear regression analysis. Based on that, a multiple linear regression model was built including all of these factors known before the start of conditioning. Smoking, diabetes, hypertension, and hyperlipidemia did not remain significant; thus, the strongest model was built including PLC at admission < 100 × 10^9^/L (ß = 0.241, p < 0.001), hemoglobin at admission < 100 g/L (ß = 0.073, p = 0.013), PTT at admission < 70% (ß = 0.103, p > 0.001), age > 60 years (ß = 0.111, p < 0.001), and HCT-CI ≥3 (ß = 0.084, p = 0.004). The R² for the overall model was 0.113 (adjusted R² = 0.109), indicating a weak goodness-of-fit according to Cohen. The model showed no autocorrelation as the value of the Durbin–Watson statistic was 1.698. An increased risk for BLEDs by a combination of these factors including PLC at admission < 100 × 10^9^/L, hemoglobin at admission < 100 g/L, PTT at admission < 70%, age > 60 years, and HCT-CI ≥3 was excluded by another binomial regression analysis using a sum score of these factors. The OR for this sum score was 1.194 (95% CI, [0.731, 19.52], p = 0.479).

**Table 2:** Transfusion requirements, onset, and duration of thrombocytopenia.

**Characteristics**	All	ASA cohort	Control cohort	p-value
Transfusion requirements				
**PRBC units, mean ± SD (range)** **No PRBC received, N (%)** **1–2 U, N (%)** **>2 U, N (%)**	0.69 ± 1.39 (0-15) 789 (70.9%) 265 (23.8%) 59 (5.3%)	0.86 ± 1.31 (0–4) 37 (64.9%) 14 (24.6%) 6 (10.5%)	0.68 ± 1.40 (0–15) 752 (71.2%) 251 (23.7%) 53 (5.1%)	0.354
**PC units, mean ± SD (range)** **No PC received, N (%)** **1–2 U, N (%)** **>2 U, N (%)**	0.8 ± 0.998 (0–12) 477 (42.9%) 580 (52.1%) 56 (5%)	1.32 ± 1.42 (0–7) 15 (26.3%) 34 (59.7%) 8 (14%)	0.78 ± 0.96 (0–12) 462 (43.8%) 546 (51.7%) 48 (4.5%)	0.007
Platelet course				
**Onset day PLC < 50 × 10^9^/L, mean ± SD (range)**	6.19 ± 1.50 (-3 - 11)	6.04 ± 1.18 (3–11)	6.20 ± 1.52 (-3–11)	0.308
**Onset day PLC < 20 × 10^9^/L, mean ± SD (range)**	7.42 ± 2.01 (-2–13)	7.42 ± 1.16 (4-11)	7.42 ± 2.05 (-2–13)	0.997
**Onset day PLC < 10 × 10^9^/L, mean ± SD (range)**	5.08 ± 4.15 (0 -14)	5.32 ± 3.86 (0–9)	5.07 ± 4.16 (0–14)	0.637
**Duration of thrombocytopenia < 50 × 10^9^/L, mean ± SD (range)**	6.92 ± 2.96 (2 - 31)	7.44 ± 2.79 (4–15)	6.89 ± 2.97 (2–31)	0.174
**Duration of thrombocytopenia < 20 × 10^9^/L, mean ± SD (range)**	2.91 ± 2.05 (0 - 23)	3.3 ± 2.22 (1–13)	2.89 ± 2.04 (0–23)	0.146
**Duration of thrombocytopenia < 10 × 10^9^/L, mean ± SD (range)**	0.89 ± 1.02 (0 - 10)	1.05 ± 1.54 (0–10)	0.88 ± 0.99 (0–9)	0.223
**Day PLC recovery >20 × 10^9^/L, mean ± SD (range)**	11.33 ± 2.75 (1 - 31)	11.71 ± 2.05 (9 - 21)	11.31 ± 2.97 (1–31)	0.277

PRBC, packed red blood cells; PC, platelet concentrate; SD, standard deviation; U, unit; PLC, platelet count.

### Description of major cardiovascular event

Given the high frequency of the CV risk factor within our cohort, we assessed the incidence of MACE during the hospital stay. Three patients suffered a MACE. Two MACEs were likely associated with the transfusion of stem cells or a serious infection and are therefore discussed in the **Supplementary Material** (1.2 **Supplementary Results and Discussion**, 1.2.1 Case discussions on patients presenting with MACE, and **Supplementary Table S3**. Cardiac comorbidities and risk factors in patients below versus above 60 years of age). Nonetheless, one patient was found unresponsive due to ventricular fibrillation and was successfully resuscitated. Subsequent coronary angiography revealed a significant occlusion of the left anterior descending artery (LAD), global hypokinesia, and reduced left ventricular ejection fraction. A drug-eluting stent and a single-chamber implantable cardiac defibrillator were implanted. The patient recovered with no obvious deficit. As CV risk factors, type II diabetes and hypertension were known prior to this event.

## Discussion

No evidence-based approach on how to handle APT during chemotherapy-induced thrombocytopenia exists, although a recent expert consensus recommended to withhold ASA with thrombocytopenia CTCAE III° unless severe CV comorbidities are present (26). In our analysis, we approached this question by assessing BLEDs and ASA discontinuation strategies in a large single-center cohort of patients with multiple myeloma undergoing ASCT. To our knowledge, this is the first study with a systematic evaluation of APT during thrombocytopenia.

The PLADO trial (14), which is the largest (prospective) trial assessing bleeding risk, included only 378 patients undergoing ASCT. Thus, we assessed the largest and most homogenous patient sample for BLEDs during ASCT so far and present true real-world data.

### Strategies for preventive acetylsalicylic acid use during autologous stem-cell transplantation

ASA represents the gold standard for preventing MACE (5). Despite its net benefit, major BLEDs were increased in both primary and secondary prevention trials (RR 2.69 [99% CI 1.25–5, p = 0.01) (5). Several retrospective analyses of cancer patients with thrombocytopenia and acute coronary syndrome suggested ASA and even dual APT to be safe and advantageous in regard to cardiac outcomes with a little risk of major bleeding (4, 27–30).

In our cohort, ASA was discontinued during thrombocytopenia of various degrees after high-dose chemotherapy. During aplasia, no or only a small percentage of new platelets are generated. Therefore, the remaining platelets were assumed to be continuously inhibited by ASA. The *in vitro* reversal of platelet inhibition in patients taking ASA was shown to be achieved by mixing inhibited platelets with 30% untreated donor platelets (31). The restoration of platelet function is mediated by TXA2 from (donor) platelets, which activates the thromboxane receptor of ASA-inhibited (host) platelets (31). This concept was supported by small case series and proof-of-principal studies *in vivo* (32–34). Despite, clinical outcome data on PLTX in patients taking ASA are scarce and the data from thrombocytopenic patients are not available at all. In our cohort, >70% of cases with ASA intake received only 0–1 unit PLTX after ASA pause during aplasia; thus, a relevant impact on continued ASA efficacy seems unlikely. Of note, if PLTX is provided during continued ASA intake, the strongest platelet inhibition by ASA 60 min after oral intake (35) should be considered, depending on the pursued effect.

We observed no MACE during the discontinuation of ASA in our cohort. Large analyses that assessed the discontinuation of ASA without surgery or bleeding after long-term use for secondary prevention demonstrated a 46% higher rate of MACE (7). This translates into one additional event in 1 of every 36 patients (7). Of note, the time from discontinuation to the thrombotic event was as early as 7–30 days in most reports (8–12, 36). In our cohort, patients discontinued ASA on average for ~9 days during thrombocytopenia. Although infections are related to a prothrombotic state, this time frame might be short enough to avoid a clinically relevant increased risk of MACE. In addition, the protective impact of thrombocytopenia for MACE is difficult to estimate or measure. This is supported by the fact that neither patients who discontinued ASA before day +1 nor patients who had an indication for secondary ASA intake but did not receive it experienced an additional MACE during ASCT. The latter group included notably 2.8% (N = 30) of control cases who had an indication for ASA as secondary prophylaxis and 0.3% (N = 3) for primary prophylaxis but did not receive it. Whether these patients developed an event after discharge could not be assessed as many of those received their follow-up visits outside our center. The only patient who developed a severe MACE during ASCT had no indication for preventive ASA to our knowledge based on his chart review.

#### Association of bleeding with acetylsalicylic acid intake

We found no significant increase of BLEDs in patients taking ASA but observed a tendency toward a higher risk of bleeding. In addition, patients on ASA received significantly more PLTX although >70% received only 0–1 unit. Above this amount, the requirements for PLTX were not related to ASA intake. Despite the increased PLTX requirements, it is unlikely that BLEDs were abrogated by the more generous transfusion approach. Patients on ASA required 1.32 units of PLTX, which is still below the reported mean requirement of 2 units (37) during ASCT and underlines our strict transfusion policy. We cannot completely exclude that ASA intake leads to a higher risk of bleeding, but in none of our analysis did it reach statistical significance.

To exclude a selection bias for analyzing BLEDs, we compared the group taking ASA until at least 1 day after ASCT with those who were excluded due to ASA discontinuation prior to day +1 (**Supplementary Table S1**). Patients differed significantly with regard to chronic kidney failure. The only documented BLED in this group was a retinal hemorrhage.

As all documented retinal hemorrhages occurred in patients taking ASA, retinal hemorrhages bear the potential of long-term vision impairments. Hypertension is one potential risk factor for retinal hemorrhages and was more prevalent in the ASA group. Thus, it is important to be aware of a possible causality between ASA and this specific BLED.

### Risk factors for bleeding

Over 80% of BLEDs occurred during PLC < 50 × 10^9^/L and majority of these with a PLC < 10 × 10^9^/L. The severity of thrombocytopenia was repetitively shown not to correlate with BLEDs (38), although a PLC < 5 × 10^9^/L was demonstrated as a risk factor for BLEDs during ASCT (14). In our cohort, multivariate analysis validated the duration of PLC < 50 × 10^9^/L, not below < 10 × 10^9^/L, as a risk factor for bleeding. Although the duration of PLC < 10 × 10^9^/L might be skewed by PLTX, these results are in concordance with previous reports.

Three risk factors for bleeding remained statistically significant in multivariate analysis: a history of GI bleeding, diarrhea, and the duration of PLC < 50 × 10^9^/L. The number of patients with former GI bleeding was very low; thus, its predictive value might be overestimated. We therefore suggest to rather focus on factors that predict a prolonged duration of PLC < 50 × 10^9^/L than the direct risk factors for bleeding. Herein, a decreased bone marrow reserve (hemoglobin at admission < 100 g/L, PLC < 100 × 10^9^/L), impaired coagulation (PTT < 70% at admission), age >60 years, or HCT-CI ≥3 points are known prior to conditioning and can help to further guide physicians in assessing the indirect risk for bleeding. None of them were significantly associated with BLEDs itself in multivariate analysis (**Supplementary Figure S2A**), neither alone or as a sum score.

The recently reported risk factors for BLEDs comprised fever (HR: 1.7, 95% CI [1.3, 2.4]) (39, 40). Neither fever nor sepsis remained an independent risk factor for bleeding in the multivariate analysis of our cohort. This might be explained by the high number of infections during ASCT: 89.9% of cases developed at least one documented episode of fever/infection, and 69.6% of BLEDs occurred during an infection. Whether this constitutes a co-occurrence or a causality remains unclear, but a statistical association could not be demonstrated.

### Limitations

The incidence of BLEDs in our cohort was lower than recently reported (20, 21). In these trials, a daily structured bleeding assessment was mandatory and was documented as patient days with bleeding to account for the course of the bleeding with regard to PLTX. We documented every BLED only once; thereby, the bleeding incidence is hardly comparable. In addition, our data are based on EMRs without these structured assessments and the underreporting of mild BLEDs (e.g., mild epistaxis) is possible.

Furthermore, the number of patients on ASA was limited, although this is a large real-world cohort and the largest sample size assessing BLEDs during ASCT so far. Therefore, our results are hypothesis-generating and this question can only be answered in a randomized controlled trial. Such a trial would require a large international cooperative initiative that does not appear to be feasible (for the design and sample size calculation of such a trial, please refer to **Supplementary Material**, 1.1 **Supplementary Methods**, 1.1.2 Design of a randomized controlled trial to assess different APT strategies, and **Supplementary Figure S1**).

### Recommendations on management of antiplatelet therapy during autologous stem-cell transplantation

**Figure 4:**
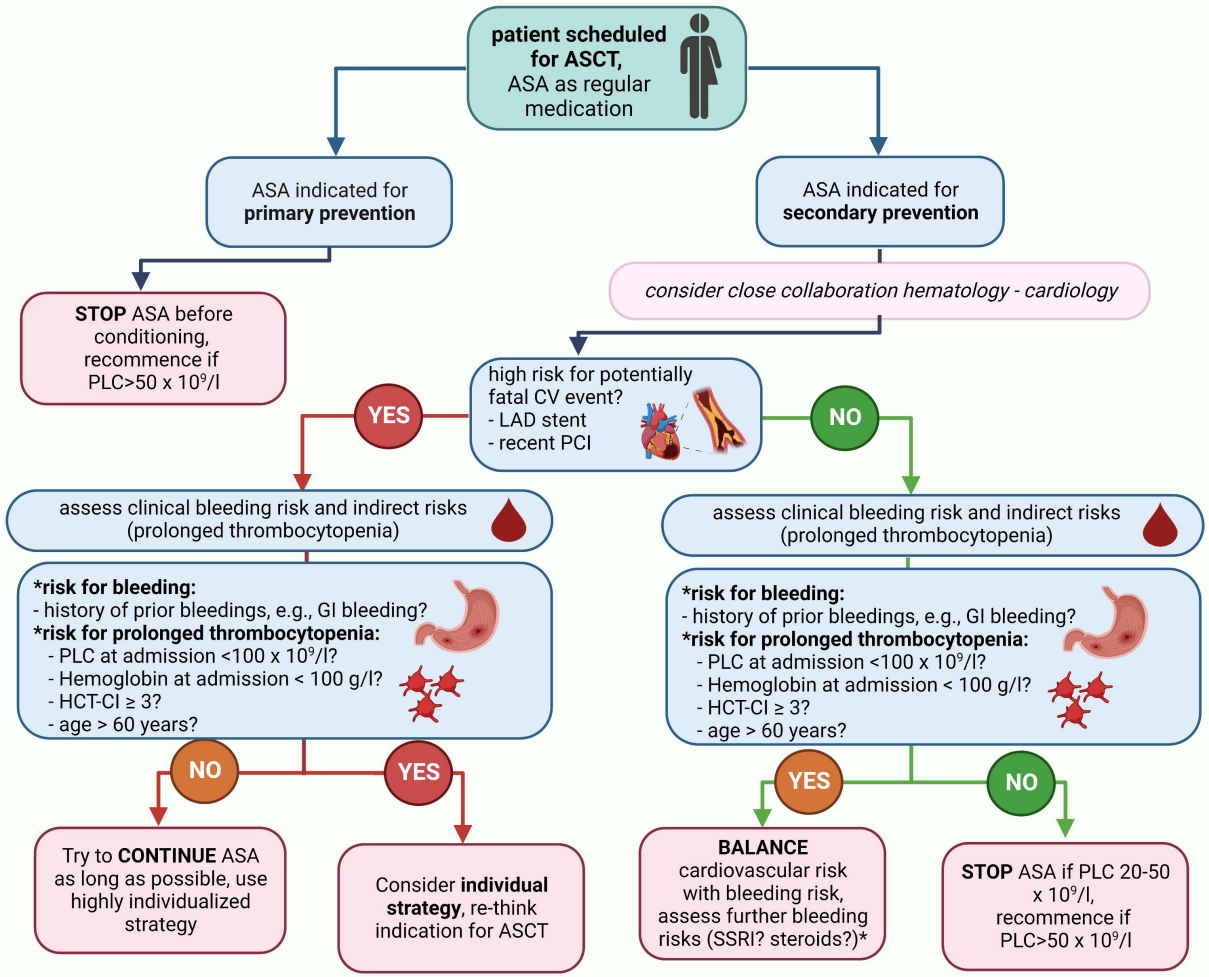
Summary of recommendations for management of APT during ASCT. If a patient is scheduled for ASCT and has an indication for APT, we recommend the depicted management strategy. *Although the use of steroids or selective serotonin reuptake inhibitors were not demonstrated to carry an additional bleeding risk in our cohort, they carry well-described bleeding risks and we would recommend to integrate considerations on their use into the individual patient management plan. *ASA, acetylsalicylic acid; CV, cardiovascular; HCT-CI, hematopoietic stem-cell transplantation comorbidity index; LAD, left-anterior descending; PCI, percutaneous coronary intervention; PLC, platelet count.*.

**Figure 5:**
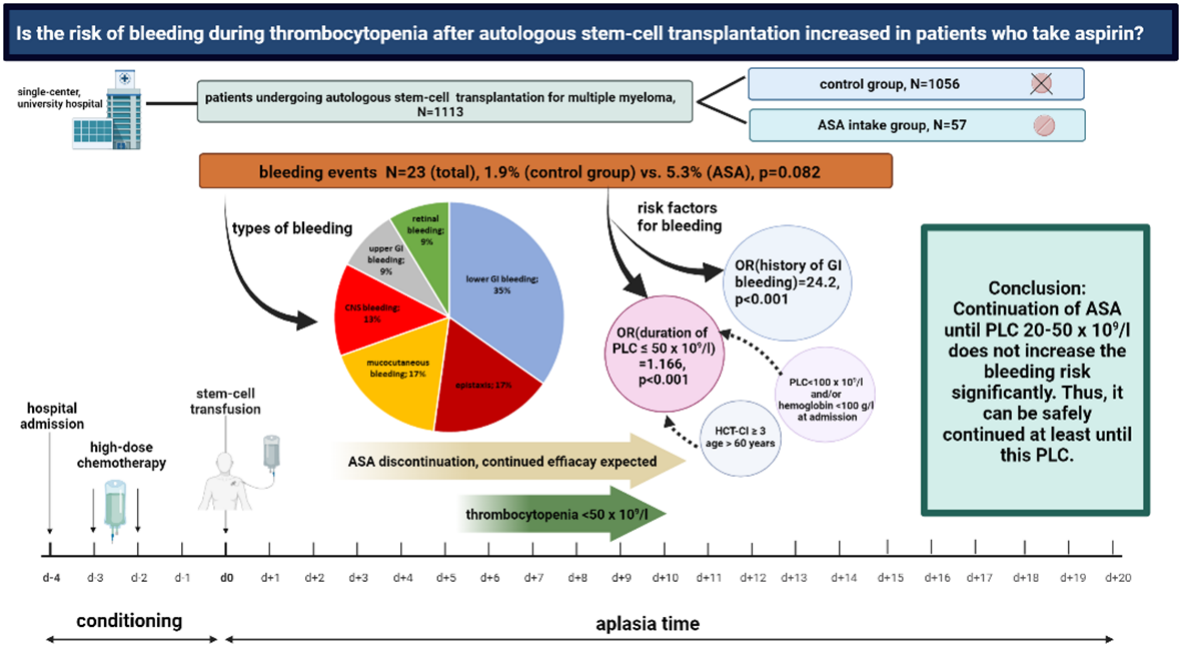
Aspirin use and bleeding events during thrombocytopenia following autologous stem-cell transplantation for multiple myeloma. Overview on the study design and major findings.

As an evidence-based strategy guided by high-quality randomized trial data will most likely never be available, the following rationale-based approach can be considered: if ASA is indicated based on a primary preventive approach, the discontinuation of ASA before conditioning can be considered based on the high number needed to treat (NNT) to prevent MACE and the relevant risk for bleeding even with normal PLC. If ASA is indicated by a secondary preventive approach, it appears safe to continue ASA until a PLC of 20–50 × 10^9^/L (CTCAE III°). We consider this PLC range rather than a fixed cut-off to pause ASA, as PLC was not measured daily in our routine clinical practice. Furthermore, a PLC range of 20–50 × 10^9^/L reflects the real-world scenario better than a fixed cut-off given the standard kinetics of thrombocytopenia during ASCT. Whether the application of ASA even during PLC < 20 × 10^9^/L with a lower transfusion trigger (e.g., 20–30 × 10^9^/L) is also safe cannot be concluded from our data as only one patient was guided with this strategy. The rationale behind such an approach is a potentially increased prothrombotic state due to a rebound effect after the discontinuation of ASA. When the ASA discontinuation strategy for the individual patient is discussed, we recommend to consider age >60 years, HCT-CI ≥3, PLC < 100 × 10^9^/L, hemoglobin < 100 g/L, and PTT < 70% at admission as predictors for a prolonged duration of PLC < 50 × 10^9^/L, and therefore, an indirect bleeding risk together with a clinical bleeding history (especially GI bleedings). Recommendations are summarized in **Figure 4**; general results are shown in **Figure 5**.

## Conclusions

Severe BLEDs are rare during ASCT even if ASA is continued until thrombocytopenia CTCAE III°. The APT in such a situation will remain an “one-size-does-not-fit-all” situation and requires a thorough balance between the individual risks for MACE and bleeding, ideally achieved by a close collaboration between hematologists and cardiologists. Especially high-risk situations for a fatal MACE (e.g., recent PCI and the involvement of LAD) might drive ASA continuation until there are very low PLCs.

## Data availability statement

The original contributions presented in the study are included in the article/**Supplementary Material**. Further inquiries can be directed to the corresponding author.

## Ethics statement

The studies involving human participants were reviewed and approved by Institutional review board of the University Heidelberg, Germany, No S-721/2018.

## Author contributions

NN participated in research design, data acquisition and analysis, and writing of the paper. BB participated in data acquisition and writing of the paper. LF participated in research design and the final editing of the paper. MK participated in data analysis and the final editing of the paper. CR participated in data analysis and the final editing of the paper. HG participated in research design and the final editing of the paper. NF participated in the final editing of the paper. CM-T participated in the final editing of the paper. KJ participated in research design and the final editing of the paper. SS participated in the final editing of the paper. MJ participated in research design, data acquisition and analysis, and the writing of the paper. All authors contributed to the article and approved the submitted version.
