# Supplementary material for: Aspirin use and bleeding events during thrombocytopenia after autologous stem-cell transplantation for multiple myeloma
Source: Front Oncol. 2023 Apr 27;13:1168120. doi: 10.3389/fonc.2023.1168120 (PMC10174307; doi:10.3389/fonc.2023.1168120)
Supplement: Supplementary file 1 [file DataSheet_1.docx]

Supplementary Material

Aspirin use and bleeding events during thrombocytopenia after autologous stem-cell transplantation for multiple myeloma

Nina Rosa Neuendorff, Boryana Boshikova, Lutz Frankenstein, Marietta Kirchner, Christian Rohde, Hartmut Goldschmidt, Norbert Frey, Carsten Müller-Tidow, Karin Jordan, Sandra Sauer, Maike Janssen*

* Correspondence: Maike Janssen, Corresponding Author: maike.janssen@med.uni-heidelberg.de

# Supplementary Data

## Supplementary Methods

### Grading of bleeding events, their definitions, and rationale

The WHO bleeding scale is the most abundant bleeding scale used for grading the severity of bleeding in hematological trials, thus, it was included for inter-trial comparability. The WHO bleeding score classifies four grades: mild (grade 1), moderate bleeding without requirements for PRBCs (grade 2); moderate to severe bleeding requiring PRBCs (grade 3), and life-threatening or limb-threatening bleeding (grade 4) (1). Nonetheless, the WHO scale has some major limitations, such as lack of formal standardization resulting in a variation of characterization and amount of reported bleeding in trials (2). Although using bleeding events ≥2° is frequently used as outcome measures, grade 2 bleeding events have never been shown to be related to significant morbidity or mortality, decreased quality of life, or grade 3 and 4 bleedings (3). Petechia and hematomas were not included in our documentation despite their inclusion in the BSMS as grade 1a/b bleeding due to the inconsistent and lacking documentation in the EMR and their probable clinical irrelevance. In addition, we graded bleeding events as 1b if no more than 1 unit of platelet transfusions were given, as 2a if > 1 unit was transfused due to bleeding despite a mild clinical course.

### Design of a randomized controlled trial to assess different APT strategies

Although the ultimate aim of ASA continuation during thrombocytopenia is to prevent MACE, the very low incidence of MACE during ASCT and the short time frame of ASA discontinuation limits its feasibility as primary endpoint for a trial. Thus, bleeding needs to be the preferred endpoint in such a trial to exclude a relevant bleeding risk associated with ASA continuation under the assumption that MACEs are prevented. To evaluate the possibility to conduct such a randomized, controlled trial to assess the bleeding risk with different modes of ASA applications, we performed a sample size calculation. Planning two independent study groups with indication for APT as secondary prevention, we would perform a 1:1 randomization between ASA discontinuation before the start of the conditioning in comparison to a discontinuation approach during PLC<20-50 x 10^9^/l (Supplementary Figure S1). Aiming at a non-inferiority approach with expected bleeding incidence of 2% (when ASA is stopped before conditioning) versus 5% (ASA continuation until PLC<20-50 x 10^9^/l) based on our data with a non-inferiority margin of -0.02, a power of 0.8, a type I error probability 0.05, and a drop-out rate of 5%, a sample size of 910 (455 in each group) would be required. Giving a proportion of patients with indication for secondary preventive ASA in 7.3 % (in our cohort) and an expected exclusion based on high risks for CVE of ~5%, ~13 090 patients needed to be screened.

## Supplementary Results and Discussions

### **Critical view on presenting cases with MACE**

Patient A presenting with a formal MACE was a 64-year-old female who developed a hypertensive crisis soon after transfusion of her autologous stem-cells on day 0 followed by a right-sided deviation of vision, monoplegia of her right arm, and confusion. On that day, her PLC was 216 x 109/l and no infection was present. cCT was unremarkable but cMRI showed a small ischemic lesion dorsally of the corpus callosum in FLAIR sequence. Symptoms resolved completely within one day. Further work-up revealed moderate plaques in both carotic arteries. No CV risk factors were known prior to ASCT. Given the timely association to the stem-cell transfusion and the hypertensive crisis which is a common side-effect of stem-cell transfusion due to cryoconserving dimethyl sulfoxide (DMSO), a primary cerebrovascular event appeared to be unlikely. A persistent foramen ovale was not explicitly excluded in the work-up as this could potentially explain the symptoms after transfusion (formation of small blood clots within the autologous stem-cell preparation that were not filtered by pulmonary capillaries).

Patient B was a 58-year-old female who experiences a complicated course of ASCT with severe pneumonia due to herpes simplex virus and pneumocystis jirovecii necessitating mechanical ventilation and a prolonged stay on intensive care unit. After respiratory recovery on day +75, she developed typical angina accompanied by shortness of breath and rise in Troponin T up to 233 pg/ml. Her electrocardiogram was unremarkable. Due to her overall reduced condition, no coronary angiography was performed and dual APT was started with the assumption of a non-ST segment elevation myocardial infarction. Her symptoms resolved within days. APT was continued but stopped after years without any further angina. No CVE was documented in her follow-up notes for ~8 years. Neither a coronary angiography nor a non-invasive evaluation of her coronary arteries was performed. Despite the typical clinical presentation of angina, the long event-free survival (even after termination of APT) and the clinical presentation in/shortly after a critical illness situation make the diagnosis of NSTEMI very unlikely. Though, there is no possibility to formally proof this hypothesis.

Patient C was already discussed within the main text and represents in our view the only real MACE during ASCT in our cohort.

**References**

1. Hunter R. World Health Organization (Who) Handbook for Reporting Results of Cancer Treatment. *WHO, Geneva, Switzerland* (1979).

2. Estcourt LJ, Heddle N, Kaufman R, McCullough J, Murphy MF, Slichter S, et al. The Challenges of Measuring Bleeding Outcomes in Clinical Trials of Platelet Transfusions. *Transfusion* (2013) 53(7):1531-43. doi: <https://doi.org/10.1111/trf.12058>.

3. Tay J, Allan D, Beattie S, Bredeson C, Fergusson D, Maze D, et al. Rationale and Design of Platelet Transfusions in Haematopoietic Stem Cell Transplantation: The Path Pilot Study. *BMJ Open* (2016) 6(10):e013483. Epub 2016/11/01. doi: 10.1136/bmjopen-2016-013483.

# Supplementary Figures and Tables

## Supplementary Figures

**Supplementary Figure S1. Suggested designs of a clinical trial assessing bleeding risks upon ASA intake**


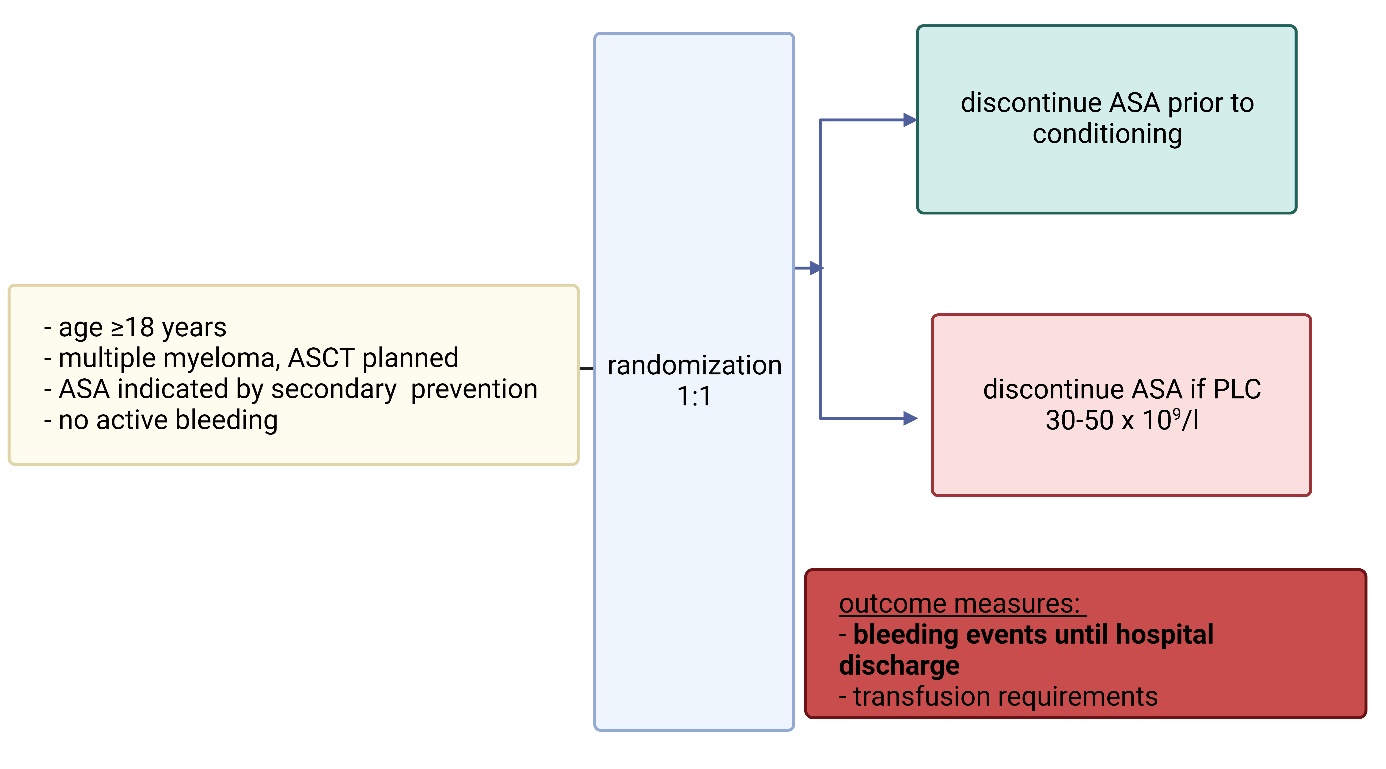


## Supplementary Figure S2. Factors associated with bleeding events and duration of PLC<10 x10^9^/l


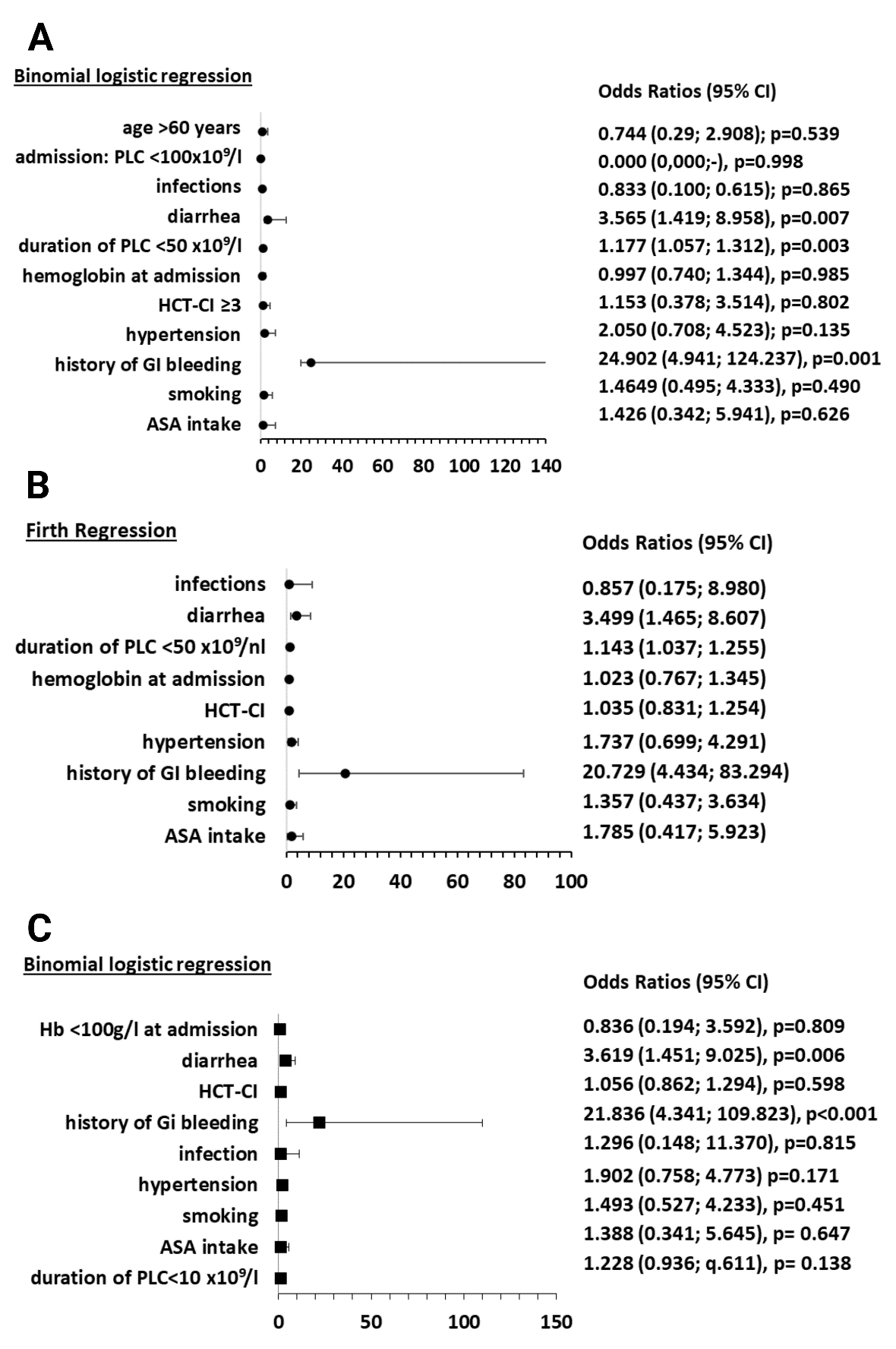


A Binomial logistic regression model (full model) on factors associated with bleeding events. This binomial logistic regression model was statistically significant, χ²(11)=38.749, p<0.001. Goodness-of-fit as assessed using Hosmer-Lemeshow-Test indicated a good model fit, χ²(8)=12.015, p>0.05. Correlations between predictor variables were low (r<0.70), indicating that multicollinearity was not a confounding factor in the analysis.

B Firth regression for factors associated with bleeding events.

C Binomial logistic regression model (full model) on factors associated with bleeding events. Platelet count (PLC) <10 x 10^9^/l was included in the model instead of PLC<50 x 10^9^/l. Note that the duration of PLC<10 x 10^9^/l could be biased by platelet transfusions. This binomial logistic regression model was statistically significant, χ²(9)=30.094, p<0.001. Goodness-of-fit as assessed using Hosmer-Lemeshow-Test indicated a good model fit, χ²(8)=9.963, p>0.05. Correlations between predictor variables were low (r<0.70), indicating that multicollinearity was not a confounding factor in the analysis.

## Supplementary Tables

| Supplementary Table S1. Characteristics of patients with probably non-efficient ASA intake and comparison to efficient ASA intake cases | | | | |
| --- | --- | --- | --- | --- |
| Characteristics | All ASA patients (N=70) | ASA efficacy assumed (N=57) | ASA stopped before day +1 (N=13) | p-value |
| ASA indication  Primary prevention  Secondary prevention | 7 (10%)  63 (90%) | 5 (8.8%)  52 (91.2%) | 2 (15.4%)  11 (84.6%) | 0.47 |
| Age [years], mean ± SD (range)  ≥65 years, N (%) | 61.53 ± 7.74 (36-75)  31 (44.3%) | 61.12 ± 8.07 (36-75)  26 (45.6%) | 63.31 ± 6.06 (50.74)  5 (38.5%) | 0.36  0.63 |
| Female, N (%) | 12 (17.1%) | 11 (19.3%) | 1 (7.7%) | 0.31 |
| HCT-CI, mean ± SD (range)  ≥3, N (%) | 2.86 ± 1.83 (0-7)  38 (54.3%) | 2.95 ± 1.9 (0-7)  31 (54.4%) | 2.46 ± 1.26 (0-4)  7 (53.8%) | 0.39  0.97 |
|  | | | | |
| First ASCT, N (%) | 52 (74.3%) | 41 (71.9%) | 11 (84.6%) | 0.34 |
| Second ASCT, N (%) | 18 (25.7%) | 16 (28.1%) | 2 (15.4%) | 0.34 |
| Melphalan dosage  200 mg/m², N (%)  100-140 mg/m², N (%) | 68 (98.6%)  1 (1.4%) | 56 (100%)  0 (0%) | 12 (92.3%)  1 (7.7%) | 0.037  0.037 |
| Transfused CD34+ cells x10^6^/kg, mean ± SD (range) | 368.31 ± 134.36 (202-836) | 365.93 ± 130.73 (202-836) | 378.77 ± 154.61 (210-727) | 0.75 |
| *Laboratory measurements at admission* | | | | |
| Hemoglobin [g/l], mean±SD (range)  <80g/l, N (%) | 117 ± 14.5 (76 – 157)  1 (1.4%) | 118.6 ± 13.6 (90-157)  0 (0%) | 109.9 ± 16.8 (76-140)  1 (7.7%) | 0.05  0.035 |
| Platelets/nl, mean±SD (range)  <100 x 10^9^/l, N (%) | 234.94 ± 78.66 (71-607)  1 (1.4%) | 234.39 ± 74.6 (135-607)  0 (0%) | 237.38 ± 98.0 (71-458)  1 (7.7%) | 0.90  0.035 |
|  |  |  |  |  |
| Chronic kidney insufficiency | 13 (18.6%) | 7 (12.3%) | 6 (46.2%) | 0.005 |
|  | | | | |
| History of GI bleeding, N (%) | 3 (4.3%) | 3 (5.3%) | 0 (0%) | 0.398 |
| History of CNS bleeding, N (%) | 0 (0%) |  |  |  |
| History of Retina bleeding, N (%) | 0 (0%) |  |  |  |
| *Cardiac comorbidities and risk factors* | | | | |
| CAD | 45 (64.3%) | 36 (63.2%) | 9 (69.2%) | 0.68 |
| AF | 3 (4.3%) | 2 (3.5%) | 1 (7.7%) | 0.50 |
| PAD | 12 (17.1%) | 11 (19.3%) | 1 (7.7%) | 0.31 |
| TIA | 1 (1.4%) | 1 (1.8%) | 0 (0%) | 0.63 |
| Stroke | 10 (14.3%) | 8 (14%) | 2 (15.4%) | 0.90 |
| PFO | 1 (1.4%) | 0 (0%) | 1 (7.7%) | 0.03 |
| CRAO | 2 (2.9%) | 2 (3.5%) | 0 (0%) | 0.49 |
| HF | 11 (15.7%) | 8 (14%) | 3 (23.1%) | 0.41 |
| TE  DVT  PE | 1 (1.4%)  1 (1.4%) | 1 (1.8%)  1 (1.8%) | 0 (0%)  0 (0%) | 0.63  0.63 |
| OSA | 1 (1.4%) | 1 (1.8%) | 0 (0%) | 0.63 |
| Hyperlipidemia | 29 (41.4%) | 25 (43.9%) | 4 (30.8%) | 0.38 |
| Diabetes mellitus | 18 (25.7%) | 12 (21.1%) | 6 (46.2%) | 0.06 |
| History of smoking  Active smoking | 17 (24.6%)  5 (7.1%) | 16 (28.6%)  5 (8.8%) | 1 (7.7%)  0 (0%) | 0.11  0.26 |
| Hypertension | 45 (64.3%) | 35 (61.4%) | 10 (76.9%) | 0.29 |
| Abbreviations: CAD, coronary artery disease; AF, atrial fibrillation; PAD, peripheral artery disease; TIA, transient ischemic attack; PFO, patent foramen ovale; CRAO, central retinal artery occlusion; PAH, pulmonary arterial hypertension; HF, heart failure; TE, thromboembolic event; DVT, deep vein thrombosis; PE, pulmonary embolism; OSA, Obstructive sleep apnea. | | | | |

| **Supplementary Table S2. Case summaries of patients with bleeding events** | | | | | | | | | | | | | |
| --- | --- | --- | --- | --- | --- | --- | --- | --- | --- | --- | --- | --- | --- |
| Number | BLED | BSMS | WHO | CTCAE  (grade) | BLED (day) | PLC count at BE onset  [10^9^/l] | Impaired coagula-tion | ASA | BLED during infection | History  of bleeding | Diagnostic studies/ interven-tions | Transfusions requirements  during bleeding | Bleeding sequelae |
| 1 | lower GI | 2a | 2 | 1 | +16 | 61 | N | N | Y | N | procto-rectoscopy | N | N |
| 2 | lower GI, hematuria | 2b | 4 | (3-)4 | +14 | 37 | N | N | Y | N | N | 10 U PRBC,  4 U PC | N |
| 3 | lower GI (hemorrhoidal) | 1b | 2 | 1 | +3 | 7 | N | N | Y | N | N | N | N |
| 4 | lower GI (rectal) | 2a | 3 | 2 | +6 | 52 | N | N | Y | N | N | 2 U PRBC | N |
| 5 | lower GI | 2a | 2 | 1 | +8 | 20 | N | N | Y | N | N | 3 U PC | N |
| 6 | lower GI,  hematuria | 2b | 3 | 3 | +6 | 16 | aPTT 34s↑  INR 1.19↑  No PPI | N | Y | N | N | 4 U PRBC,  3 U PC | N |
| 7 | lower GI (hemorroidal) | 1b | 2 | 1 | +4 | 160 | N | N | N | N | N | N | N |
| 8 | lower GI | 1b | 2 | 1 | +5 | 24 | N | N | Y | GI blee-ding | N | 1 U PC | N |
| 9 | upper GI (melaena) | 1b | 2 | 1 | +7 | 10 | N | N | N | N | N | 1 U PC | N |
| 10 | Epistaxis, probably leading to melaena | 2a | 3 | 3 | +2 | 4 | N | N | Y | N | ENT consult, nasal packing | 2 U PRBC  2 U PC | N |
| 11 | upper GI  (Mallory-Weiss-lesions) | 2a | 3 | 3 | +5 | 39 | N | N | N | N | gastroscopy | 2 U PRBC,  1 U TC | N |
| 12 | CNS  (multifocal ICH) | 2b | 2 | 1 | +40 | 7 | N | N | Y | N | CCT, CMRI neurology consultation | 2 U PC | ↓ |
| 13 | CNS (SAH, ICH of cerebellum) | 2b | 2 | 1 | +10 | 10 | aPTT 35s↑ | N | Y | N | CCT, CMRI  neurology consultation | 1 U PC | ? |
| 14 | CNS, epistaxis | 2b | 3 | 1 | +8 | 9 | aPTT 35s↑ | N | Y | N | CCT (x3)  neurology consultation | 5 U PC,  2 U PRBC | ? |
| 15 | retinal | 1b | 2 | 1 | +6 | 5 | N | Y, EE, AS: PLT<50/nl | N | N | ophthalmology consult, no loss of vision, no intervention | N | N |
| 16 | retinal | 2b | 4 | 1 | -2 | 179 | no | Y, EE, AS: PLT<50/nl | N | GI bleeding | CCT, intermittent impaired vision | N | ? |
| *17* | *retinal* | *1b* | *2* | *2* | *+12* | *14* | *no* | *Y, no EE; AS: stopped at admission* | *Y, during E. coli sepsis and hypertensive crisis* | *N* | *(CCT)* | *N* | *N* |
| 18 | hematuria  (mucocuta-neous) | 2a | 2 | 1 | +8 | 18 | N | N | Y | N | urology consult | 1 U PC | N |
| 19 | epistaxis | 2a | 3 | 3 | +6 | 6 | N | N | N | N | N | 4 PRBC,  1 PC | N |
| 20 | epistaxis | 2a | 2 | 3 | +9 | 5 | N | Y, EE | Y | N | ENT consult, nasal packing | 3 U PC | N |
| 21 | epistaxis | 2a | 2 | 2 | +10 | 10 | aPTT 39s↑  INR 1.19↑ | N | Y | N | ENT consult, nasal packing | 1 U PC | N |
| 22 | mucocutaneous | 1b | 2 | 1 | +4 | 7 | N | N | N | N | N | 1 U PC | N |
| 23 | mucocutaneous | 2a | 2 | 1 | +2 | 6 | N | N | Y | N | N | 3 U PC | N |
| 24 | mucocutaneous | 1b | 2 | 1 | +8 | 9 | N | N | Y | N | N | 1 U PC | N |
| Abbreviation: aPTT, activated partial thromboplastin times; AS, ASA strategy; BLED, bleeding event; BSMS, Bleeding Severity Measurement Scale, CCT, cranial computed tomography; CMRI, cranial magnetic resonance imaging; CTCAE, Common Terminology Criteria for Adverse Events; EE, efficacy of ASA expected; ENT, ear, nose and throat; ICH, intracranial hemorrhage; INR, International normalized ratio of prothrombin time; PC, platelet concentrate; PLC, platelet count; PRBC, packed red blood cells; SAH, subarachnoid hemorrhage; U, unit; WHO, World health organization bleeding scale.  *this case was excluded from analysis as ASA was stopped before start of conditioning | | | | | | | | | | | | | |

| Supplementary Table S3. Cardiac comorbidities and risk factors in patients below versus above 60 years of age | | | |
| --- | --- | --- | --- |
| Characteristics (N=1126*) | Pts < 60 years (N=543, 48.2%) | Pts ≥ 60 years (N=583, 51.8%) | p-value |
| CAD | 31 (5.7%) | 48 (8.2%) | 0.103 |
| AF | 7 (1.3%) | 25 (4.3%) | 0.003 |
| PAD | 2 (0.4%) | 11 (1.9%) | 0.023 |
| TIA | 1 (0.2%) | 4 (0.7%) | 0.375 |
| Stroke | 4 (0.7%) | 8 (1.4%) | 0.389 |
| PFO | 0 (0%) | 5 (0.9%) | 0.063 |
| CRAO | 0 (0%) | 5 (0.9%) | 0.063 |
| HF | 23 (4.2%) | 14 (2.4%) | 0.095 |
| TE  DVT  PE | 38 (7%)  16 (2.9%) | 37 (6.3%)  17 (2.9%) | 0.720  1 |
| OSA | 5 (0.9%) | 18 (3.1%) | 0.011 |
| Hyperlipidemia | 16 (2.9%) | 51 (8.7%) | <0.001 |
| Diabetes mellitus | 36 (6.6%) | 66 (11.3%) | 0.007 |
| History of smoking  Active smoking | 84 (15.5%)  31 (5.7%) | 65 (11.2%)  14 (2.4%) | 0.035  0.006 |
| Hypertension | 114 (21%) | 231 (39.6%) | <0.001 |
| Abbreviations: CAD, coronary artery disease; AF, atrial fibrillation; PAD, peripheral artery disease; TIA, transient ischemic attack; PFO, patent foramen ovale; CRAO, central retinal artery occlusion; PAH, pulmonary arterial hypertension; HF, heart failure; TE, thromboembolic event; DVT, deep vein thrombosis; PE, pulmonary embolism; pts, patients; OSA, Obstructive sleep apnea.  *all patients including those without efficient ASA intake were included into this overview | | | |
